# Natal origin of Pacific bluefin tuna *Thunnus orientalis* determined by SIMS oxygen isotope analysis of otoliths

**DOI:** 10.1371/journal.pone.0272850

**Published:** 2022-08-10

**Authors:** Yulina Hane, Takayuki Ushikubo, Yusuke Yokoyama, Yosuke Miyairi, Shingo Kimura

**Affiliations:** 1 Graduate School of Frontier Sciences, The University of Tokyo, Kashiwa, Chiba, Japan; 2 Atmosphere and Ocean Research Institute, The University of Tokyo, Kashiwa, Chiba, Japan; 3 Kochi Institute for Core Sample Research, Japan Agency for Marine-Earth Science and Technology, Nankoku, Kochi, Japan; 4 Department of Earth and Planetary Science, Graduate School of Science, The University of Tokyo, Hongo, Tokyo, Japan; 5 Organization for Programs on Environmental Sciences, Graduate School of Arts and Sciences, The University of Tokyo, Komaba, Tokyo, Japan; CSIR-National Institute of Oceanography, INDIA

## Abstract

Accurate understanding of changing population dynamics associated with climate change is critical for managing highly migratory fish species. However, long-term data on population dynamics and the resulting recruitment variability is still lacking for many species, making it difficult to predict and integrate the effects of ocean warming into management schemes. In this study, high-resolution stable oxygen isotope (δ^18^O) analysis was performed on the otoliths of adult Pacific bluefin tuna *Thunnus orientalis* using secondary ion mass spectrometry (SIMS) to determine the natal origin of an individual fish. The core δ^18^O_otolith_ corresponding to the larval stage greatly varied among the individuals, indicating that the larvae experienced a wide range of thermal environments. The non-hierarchical cluster analysis performed on the core δ^18^O_otolith_ grouped fish into those with higher δ^18^O_otolith_ (lower temperature) and those with lower δ^18^O_otolith_ (higher temperature), most likely representing relative temperature difference experienced between fish born in the Sea of Japan and in the Nansei Islands area. The Nansei Islands area cluster showed more variability in the early otolith growth indicating a longer spawning season, which is consistent with the observed longer spawning duration in this area. The absolute temperature estimates based on the SIMS-measured core δ^18^O_otolith_ were significantly higher than those expected from sea surface temperature data, suggesting the effects of matrix-related bias on the temperature offsets. The relative temperature difference, however, matched well with the known spawning temperature range of the two spawning grounds. The recruitment contribution from each spawning ground (all year-classes pooled, *n* = 51) was 45% in the Sea of Japan and 55% in the Nansei Islands area. Overall, this study demonstrated the effectiveness of SIMS δ^18^O_otolith_ analysis for investigating the natal origin of fish and its potential application in fish population dynamics studies.

## Introduction

Population mixing is a common phenomenon observed among various marine fish species where fish originating from geographically distinct spawning grounds mix as they grow and migrate to the common nursery and feeding habitats [1, 2]. While mixing occurs at various levels depending on the species, the extent to which population dynamics is affected by mixing is unknown for most species, causing great uncertainty in the stock assessment and management. Highly migratory species such as tunas migrate long distances over thousands of kilometers across oceans, often crossing international borders during their life cycle [3]. Such long-distance migration and the resulting population connectivity complicate management because they are caught by multiple countries where the natal origin of fish is not being monitored. Neglecting spatial population dynamics and structure may result in failure to accurately assess stock status and consequently promote ineffective fisheries management measures [4]. It is thus important to properly assign the natal origin of fish to better understand population dynamics of marine organisms as such knowledge is key to effective stock assessment and management for many species [5–7].

**Fig 1:**
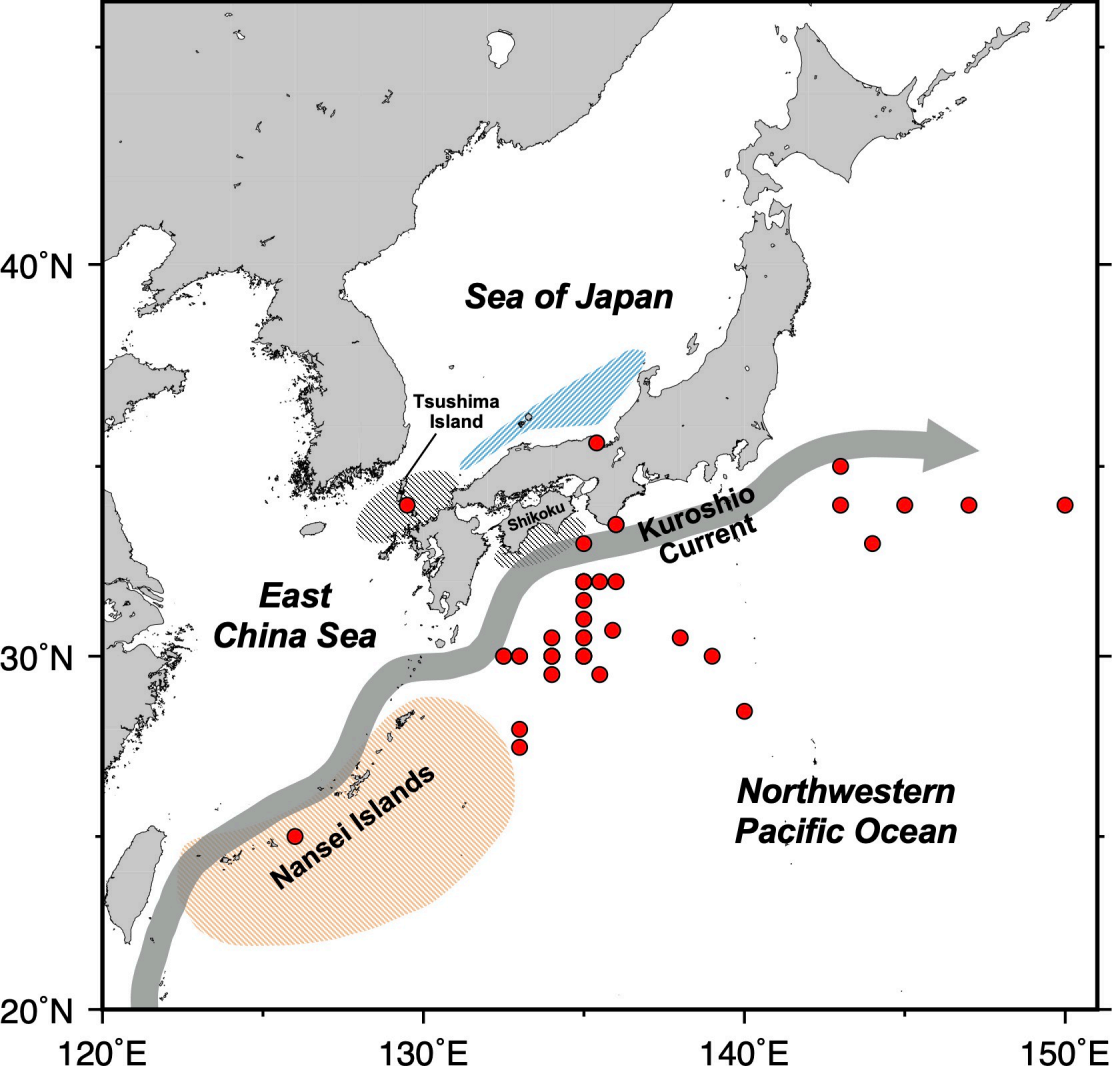
Map showing two major spawning grounds of Pacific bluefin tuna. Colors represent the spawning ground in the southwest of the Sea of Japan (blue shade) and the Nansei Islands area (orange shade). Juvenile nursery areas are shown in the black-shaded areas. Red circles indicate catch locations of the fish samples used in this study.

Pacific bluefin tuna (PBT, *Thunnus orientalis*) is one of the most commercially important tuna species harvested around the world whose annual total harvest value estimated to be US $207 million in 2020 (Japan [8] and the US [9] combined). PBT has been listed as a near threatened species on the IUCN Red List [10] with over 96% of the original biomass being depleted primarily due to overfishing [11]. PBT is a highly migratory species that is currently managed based on the single stock hypothesis [12]. PBT has two distinct seasonal spawning grounds in the western North Pacific. One is located in waters off the Nansei Islands stretching between Kyushu and Taiwan (hereafter termed “the Nansei Islands area”) and the other is located in the southwestern part of the Sea of Japan (Fig 1). Spawning in the Nansei Islands area occurs earlier and longer from late April to early July at temperatures between 26 and 29°C [13–15], whereas that in the Sea of Japan occurs during July and early August at temperatures greater than 20°C [16]. Fish hatched in the Nansei Islands area are transported northward by the Kuroshio Current and enter the coastal waters off the Tsushima Island in the East China Sea or Shikoku in the Pacific [17], where they spend their first summer as juveniles. Fish hatched in the Sea of Japan mainly feed and migrate in the Sea of Japan during their first year [18] and mix with fish born in the Nansei Islands area. Some portions of young PBT (ages 0.5 to 2) initiate trans-Pacific migrations from the western Pacific to the eastern Pacific [19–21], crossing more than 8,000 km to reach the feeding grounds in the California Current Large Marine Ecosystem (CCLME). After spending several years in the CCLME, mature PBT return to the western Pacific to spawn [21]. Species like PBT whose spawning occurs seasonally in geographically limited areas are particularly vulnerable to ocean warming as early growth and survival of their offspring often depends primarily on water temperature [22, 23] coupled with prey availability [24], resulting in the interannual recruitment variability [25–27]. Despite its importance for proper stock assessment and management, such climate-induced variability in the recruitment of PBT from each spawning ground remains largely unknown due to difficulties in monitoring long-term responses to climate stressors.

Otoliths, or ear stones, are paired calcified aragonitic structure found in the inner ear of all teleost fish. Unlike other calcified structures such as bones and scales, otoliths are acellular and metabolically inert and therefore its chemical compositions remain stable for the entire life of the fish [28]. Stable oxygen isotope ratio (^18^O/^16^O) in otoliths (δ^18^O_otolith_) has been widely used as an environmental indicator for reconstructing water temperature and salinity histories of an individual fish [29–31]. Otoliths are generally formed at or near isotopic equilibrium with the ambient water and otolith–seawater δ^18^O fractionation has been shown to be temperature dependent [29, 32–34]. δ^18^O_otolith_ generally displays a negative correlation with water temperature, with minor differences in fractionation factors among species. Using such temperature dependence of the δ^18^O_otolith_, many studies have taken advantage of this techniques to reconstruct migration histories [35], horizontal and vertical distribution [36], and population structure [1, 25, 37] of various fish species. Previous studies have successfully distinguished the eastern (the Mediterranean Sea) and western (the Gulf of Mexico) stocks of Atlantic bluefin tuna (ABT, *Thunnus thynnus*) using differences in water temperature and salinity reflected on the δ^18^O_otolith_ values deposited during the young-of-the-year (YOY) period between nursery areas [37, 38]. Although a similar study conducted with PBT otoliths [39] has reported that the timing of δ^18^O_otolith_ enrichment could be used as one of the indicators of natal origin based on the difference in spawning season, they found no difference in the core δ^18^O_otolith_ values despite the Sea of Japan being a cooler environment relative to the Nansei Islands area. This might be due to the limited spatial resolution caused by micromilling to obtain enough otolith powder needed for the conventional isotope ratio mass spectrometry (IRMS), which inevitably results in temporal averaging of δ^18^O_otolith_ allowing analysis only beyond juvenile and immature stages. As a result, temperature differences experienced during the egg and larval stages may be masked by subsequent migration and thus cannot be detected.

In this study, we determined the natal origin of PBT using high-resolution δ^18^O_otolith_ analysis conducted by secondary ion mass spectrometry (SIMS). SIMS has proven to provide significant advantage in spatial resolution over traditional IRMS as it can analyze the otolith region as small as 5 to 15 μm (corresponding to sub-annual, seasonal, weekly, or shorter time scales depending on the otolith regions) with high precision and accuracy [40, 41]. As otoliths deposited during the egg and larval stages is very small usually less than 50 μm in size, SIMS is currently the only method that can measure the isotopic composition of the early life stages of the fish. Based on the assumption that the larvae hatched in the Sea of Japan experience relatively lower temperatures than those hatched in the Nansei Islands area, we tested our hypothesis that the core δ^18^O_otolith_ serves as a natal origin indicator of PBT. In addition, ambient water temperatures experienced during the larval stage were reconstructed using the species-specific temperature fractionation equation developed for PBT larvae [34] to examine the accuracy of temperature reconstruction using the SIMS method.

## Materials and methods

### Otolith samples

Sagittal otoliths, usually the largest of the three pairs of otoliths (i.e., sagitta, lapillus, and asteriscus) were collected from the adult PBT caught in waters around Japan (Fig 1). Most fish were caught by longlining, with some fish caught by set net and traditional stone fishing. Each fish was aged based on the number of opaque zones (dark line-like structures formed annually) and the edge condition of the otoliths, following the age determination protocol for PBT [42]. Specifically, aging was performed with two readers without prior access to any sampling or biological information on fish that might bias the reading results. Third reading was conducted when the two readings disagreed, resolving on the final age count (71% of the readings agreed between the two readers). Year class was then determined for each fish using the count of opaque zones and the catch date. The summary of sampling and biological information of the fish samples used in this study are presented in Table 1.

**Table 1:** Summary on sampling data, biological information, and δ^18^O_otolith_ analyses of Pacific bluefin tuna otoliths.

Sample ID	Catch location	Date of catch	Year class	Weight (kg)	Number of SIMS spots
T16L	Nearshore off Kii Peninsula	1 June 2015	2007	119.0	10
T57R	Nearshore off Maizuru	4 June 2016	2009	108.0	13
T59R	320 km off Kii Peninsula	2 March 2017	1997	446.0	11
T60R	29.0–32.0°N, 134.0°E	16 April 2017	2010	232.0	15
T63L	29.0–30.0°N, 134.0°E	25 April 2017	2011	126.0	11
T65R	30.0°N, 134.0°E	26 April 2017	2011	105.0	14
T68R	30.0°N, 135.0°E	26 April 2017	2011	121.0	12
T70R	30.0°N, 134.0°E	26 April 2017	2004	356.0	10
T72L	30.0°N, 133.0–135.0°E	26 April 2017	2009	121.0	10
T76R	30.0–31.0°N, 137.0–139.0°E	30 April 2017	2011	117.0	10
T77R	30.0°N, 135.0°E	30 April 2017	2011	95.4	10
T81L	32.0°N, 136.0°E	5 May 2017	2010	110.0	10
T82L	34.0°N, 143.0°E	5 May 2017	2010	104.0	14
T84L	34.0°N, 142.0–148.0°E	7 May 2017	2009	110.0	11
T85R	28.0–31.0°N, 135.0–136.0°E	7 May 2017	2010	99.6	11
T86R	34.0°N, 147.0°E	7 May 2017	2009	120.0	11
T89L	30.0°N, 132.0–133.0°E	7 May 2017	2011	105.0	11
T91L	27.0–30.0°N, 140.0°E	8 May 2017	2011	108.0	10
T98L	27.0–29.0°N, 132.0–134.0°E	12 May 2017	2009	104.0	10
T101R	34.0°N, 150.0°E	15 May 2017	2011	94.2	12
T102L	25.0°N, 126.0°E	16 May 2017	2012	128.0	11
T106L	30.0°N, 135.0°E	22 May 2017	2010	106.0	12
T108R	32.0°N, 135.0°E	16 May 2017	2009	103.0	12
T111R	29.0–31.0°N, 134.0–136.0°E	18 May 2017	2013	97.8	11
T113R	29.0–32.0°N, 134.0–136.0°E	22 May 2017	2010	106.0	10
T119L	32.0°N, 135.0°E	25 May 2017	2009	121.0	11
T120L	29.0–32.0°N,134.0–136.0°E	22 May 2017	2009	109.0	12
T122L	33.0°N, 144.0°E	2 June 2017	2009	105.0	10
T124L	32.0°N, 135.0°E	2 June 2017	2010	121.0	12
T125L	32.0°N, 135.0°E	2 June 2017	2012	105.0	9
T127R	32.0°N, 135.0°E	2 June 2017	2011	106.0	10
T128R	32.0°N, 135.0°E	26 May 2017	2010	106.0	12
T129R	35.0°N, 143.0°E	31 May 2017	2010	109.0	12
T133R	Nearshore off Kii Peninsula	28 May 2017	2011	116.0	10
T134L	Nearshore off Kii Peninsula	28 May 2017	2010	111.0	11
T139R	Nearshore off Kii Peninsula	31 May 2017	2007	120.0	10
T142L	32.0°N, 135.0°E	7 June 2017	2010	108.0	10
T143L	31.0–32.0°N, 135.0°E	2 June 2017	2010	129.0	12
T145R	28.0–32.0°N, 130.0–135.0°E	4 June 2017	2010	97.8	11
T147L	28.0–32.0°N, 131.0–135.0°E	7 June 2017	2009	97.2	10
T148L	28.0–32.0°N, 131.0–135.0°E	7 June 2017	2010	99.8	11
T150L	32.0°N, 135.0–136.0°E	7 June 2017	2010	104.0	11
T151R	33.0°N, 135.0°E	7 June 2017	2013	114.0	9
T161L	30.0°N, 134.0°E	13 April 2018	2009	237.0	10
T162L	31.0°N, 135.0°E	25 April 2018	2012	205.0	10
T164L	30.0°N, 134.0°E	30 April 2018	2012	106.0	13
T64R*	30.0°N, 139.0°E	26 April 2017	2011	146.0	78
T75R*	30.0–32.0°N, 132.0–134.0°E	30 April 2017	2013	52.2	42
T104L*	27.0–28.0°N, 132.0–134.0°E	18 May 2017	2012	99.2	53
T118R*	Nearshore off Kii Peninsula	22 May 2017	2010	138.0	44
T131R*	Nearshore off Kii Peninsula	28 May 2017	2011	118.0	42

*Samples analyzed in Hane et al. (2020) were added to increase the sample size (*n* = 51 in total).

The extracted otoliths were rinsed and cleaned with Milli-Q water to remove any remaining soft tissue, air-dried in a clean environment, and individually stored in a microtube prior to the sample preparation for SIMS analysis.

### Sample preparation for SIMS analysis

**Fig 2:**
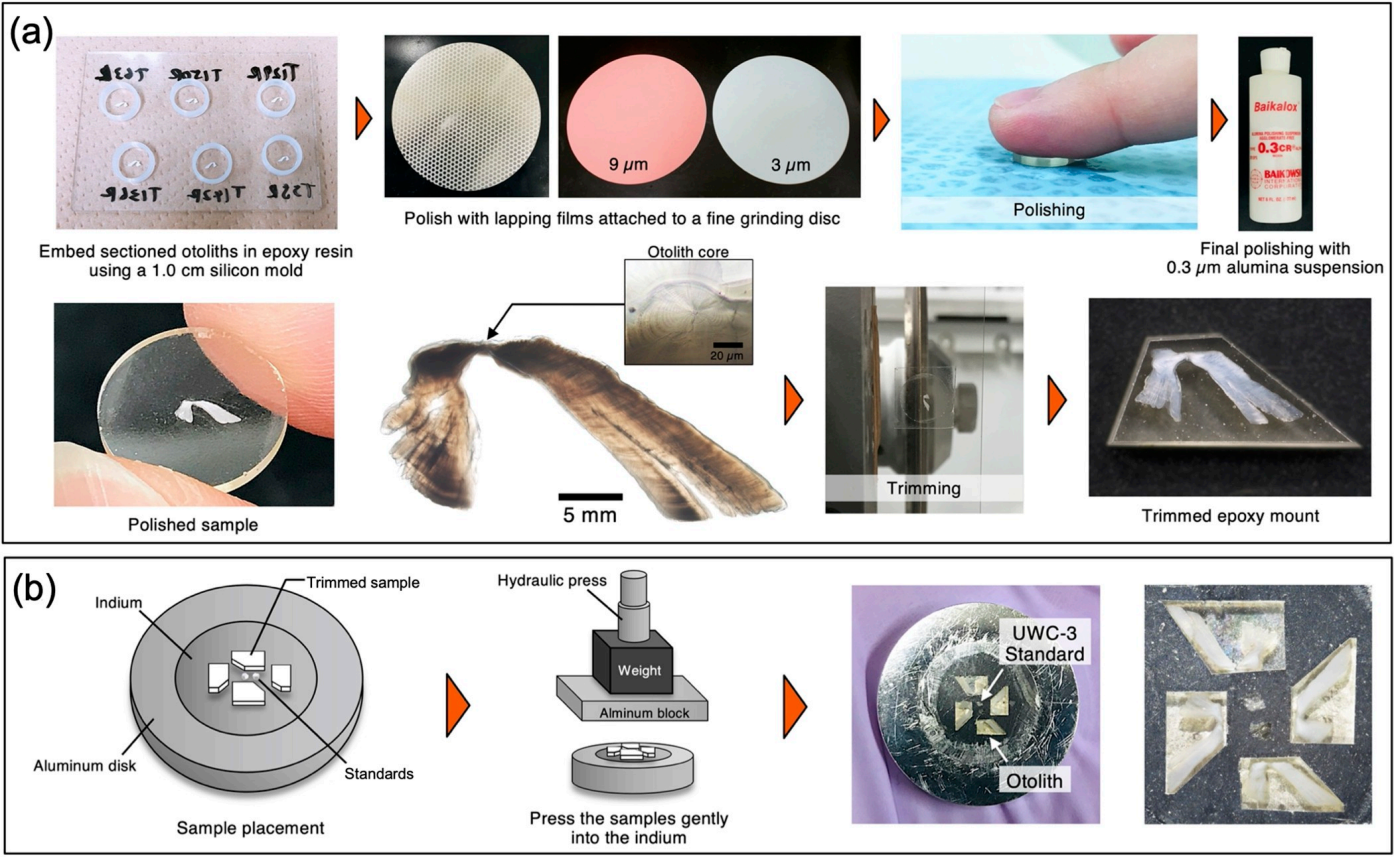
Otolith sample preparation method using indium mount for SIMS δ^18^O analysis. (a) Sectioned otoliths are embedded in epoxy resin using a 1 cm silicon mold. The back of the sample surface (non-sample side) is first ground with a grinding machine to a thickness of approximately 1.5 mm. The sample surface is then polished with 9 and 3 μm lapping film, followed by a final polishing with 0.3 alumina suspension. Finally, the excess epoxy resin around the otolith is trimmed off. (b) Two pieces of pre-polished UWC-3 standards are pressed into the center of an indium mount with a clean plastic. Samples are placed within a 1 cm diameter circle of the mount and then gently pressed into the indium using a hydraulic hand press. Pressing process should be done in a repeated manner with a force of 0.2 to 0.3 kN until the otoliths are fully embedded, and then the final press is done with 10 kN.

With carbonate samples like otoliths that require precise sectioning and polishing, only one sample can be prepared in a single epoxy mount at a time. For SIMS analysis with a large number of samples, this becomes a problem as the sample stage of currently available SIMS can accommodate only up to 6 sample mounts in the vacuum system at once, limiting the number of samples that can be analyzed per session. Indium mounts are commonly used for volatile analysis by SIMS as it contains no or a minimum amount of carbon, hydrogen, and sulfur [43] and can carry multiple samples by pressing the polished samples into the indium. Yet, they have rarely been used for carbonate samples. We thus adopted and modified this method to prepare multiple otolith thin sections in a single indium mount (Fig 2). This method greatly increases the sample throughput per session and thus allows for more efficient analysis. The presented method (Fig 2) is based, with modifications and additional procedures, on the previous sample preparation protocol developed by Hane et al. [44], which is intended for producing a mirror-finished 1-inch (2.54 cm) epoxy mount containing a standard material and a single otolith thin section of which otolith primordium is exposed on a flat surface.

Sectioning of otoliths was done according to the sample preparation protocol of Hane et al. [44]. The sectioned otolith was embedded in epoxy resin (EpoxyCure 2 Resin, Buehler) using a 1.0 cm silicon mold that was placed on a large microscope slide with a thin layer of silicon lubricant, and it was kept at room temperature for 24 h to cure the resin (Fig 2A). The epoxy disk containing the otolith section was successively polished with 9 and 3 μm lapping film attached to a stainless-steel fine grinding disk (Japanese Patent No. 6754519) until the otolith primordium was exposed on a flat surface. Final polishing was performed briefly with 0.3 μm alumina suspension (Baikalox 0.3 CR, Baikowski) to produce a mirror-polished surface. The resulting epoxy mount was then carefully attached to a microscope slide using double-sided adhesive tape and the excess epoxy resin around the otolith was trimmed off by an automatic low-speed precision cutter (IsoMet 5000, Buehler) equipped with a 0.3 mm thick diamond blade (IsoMet 15LC, Buehler).

**Fig 3:**
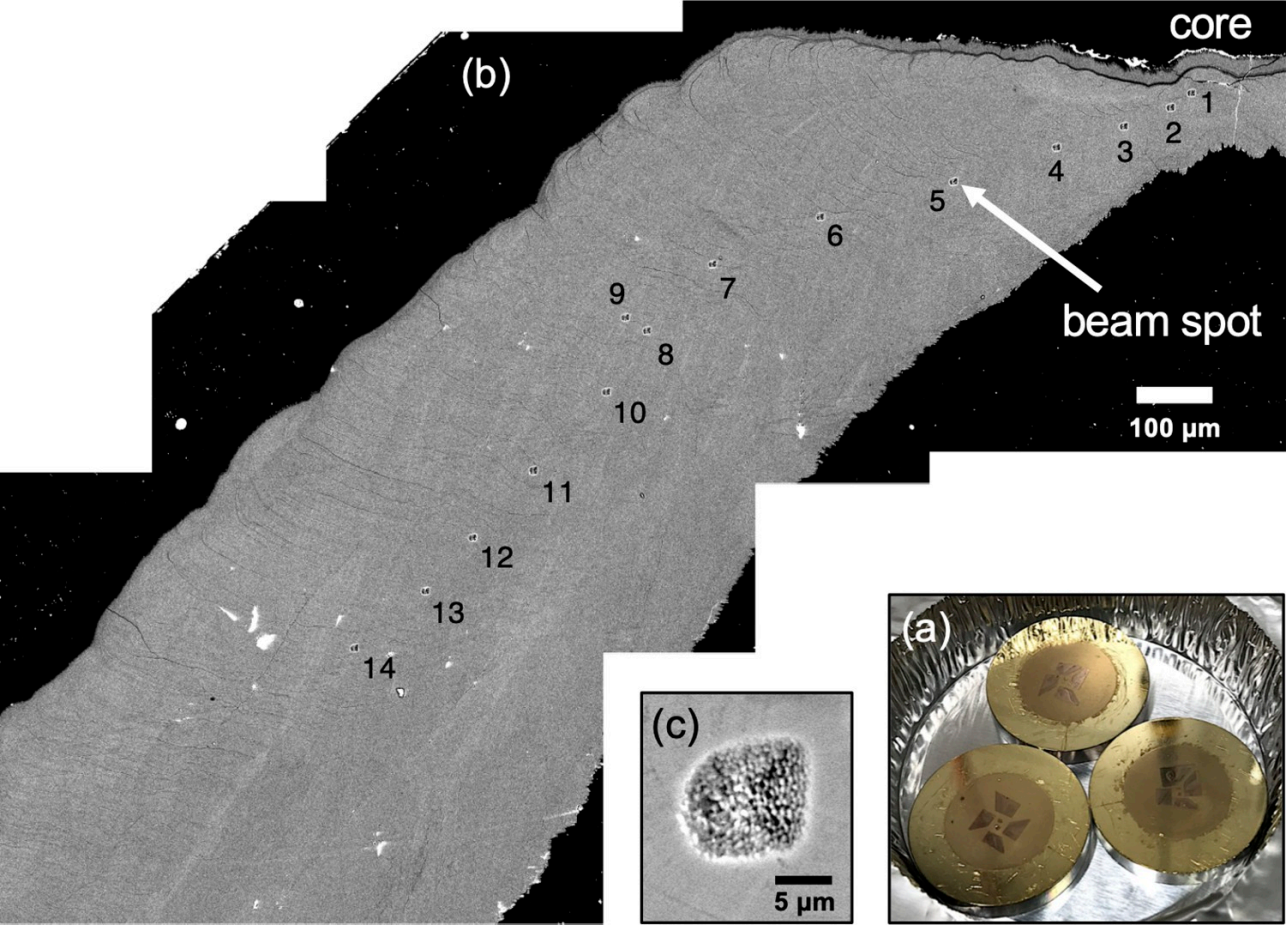
SIMS analysis of the otoliths of Pacific bluefin tuna. (a) Gold-coated indium mount containing 2 pieces of calcite UWC-3 standard and 4 otolith thin sections. (b) Cross-sectional image of an otolith taken with an electron probe micro analyzer under scanning electron microscope mode. SIMS beam spots are indicated by numbers. (c) An ion microprobe spot sputtered with a ^133^Cs^+^ primary ion beam focused to a diameter of 10 μm.

The preparation of an indium mount is described in [45] in detail. First, two pieces of pre-polished UWC-3 calcite standards (δ^18^O = 12.49‰, Vienna Standard Mean Ocean Water [VSMOW], [46]) were placed at the center of the indium mount under an optical microscope and are gently pressed into the indium with a clean piece of plastic. They do not need to be fully embedded in the indium at this point, but instead need to be stably positioned and fixed. Three to four trimmed otolith samples were placed within a 1 cm-diameter circle of the indium mount along with pre-polished UWC-3 calcite standards (Fig 2B). The samples were then gently pressed into the indium using the hydraulic hand press. To avoid breaking the samples, the pressing process should be performed successively with a force of 0.2 to 0.3 kN until the otoliths are fully embedded in the indium. After making sure the sample surface is flat with no excess indium left, a final press was done with a force of 10 kN and the indium mount was left pressed for about two to three hours before releasing the pressure. The sample surface was then polished briefly using 0.3 μm alumina suspension to remove the dust particles. Before analysis, the samples were cleaned in an ultrasonic cleaner using a Teflon beaker and dried in a vacuum oven at 40°C for 2 h. They were then sputter-coated with ~60 nm gold (Fig 3A).

### SIMS δ^18^O analysis

Oxygen stable isotope analyses were performed *in-situ* on the PBT otoliths using a CAMECA IMS-1280 large radius, multi-collector ion microprobe (SIMS) at the Kochi Institute for Core Sample, JAMSTEC, in Japan. As the peak in δ^18^O_otolith_ represents the coldest temperature experienced by fish in winter, we measured δ^18^O_otolith_ values from the otolith core until the first peak in δ^18^O_otolith_ was detected to obtain a chronological profile recording the early life and immature stages (corresponding to about 7 to 9 months old) of an individual fish (Fig 3B).

Each spot was sputtered with a ^133^Cs^+^ primary ion beam (20kV, 1.5 to 1.9 nA) focused to a spot diameter of 10 μm, resulting in a pit depth of approximately 1 μm (Fig 3C). Each analysis took about 3 min, consisting of pre-sputtering (10 s), automatic centering of the secondary ion beam (90 s), and the isotope measurements with 20 analytical cycles (40 s). The secondary ions (^16^O^–^, ^18^O^–^, and ^16^OH^–^) were accelerated at 10 kV and detected simultaneously by three Faraday cup detectors. To monitor and evaluate the relative hydrogen content contained in the otoliths, the ^16^OH^–^/^16^O^–^ ratio of each δ^18^O measurement was background-corrected by subtracting the average ^16^OH^–^/^16^O^–^ ratios of the UWC-3 bracketing analyses. The average count rates for ^16^O^–^ and ^18^O^–^ were 2.9 × 10^9^ and 5.9 × 10^6^ counts per second (cps), respectively.

Five spot measurements of UWC-3 calcite standard were made before and after every 10 to 15 unknown sample measurements to assess the spot-to-spot reproducibility of sample analyses and correct for instrumental mass fractionation. The spot-to-spot reproducibility throughout the analysis was mostly between ±0.3 to ±0.5‰ (± 2 SD). In total, 533 δ^18^O_otolith_ measurements were made on the otoliths of 46 bluefin tuna by SIMS together with 315 bracketing measurements of the UWC-3 standard. The transect length of each sample ranged from 1.02 to 1.69 mm with spot-to-spot distance ranging roughly from 50 to 200 μm. The temporal resolution of the 10 μm beam spot corresponded to 3–5 days around the otolith core based on the daily growth increment counts and several weeks to a month for the outer regions based on the relative position and ratio of the beam spot.

After analysis, each spot was imaged and observed using electron probe micro analyzer (JXA-8230, JEOL) under scanning electron microscope (SEM) mode to check for any cracks and inclusions that might bias the δ^18^O values (e.g., [47]). The secondary ion yield (^16^O^–^, cps/nA) relative to the mean of the UWC-3 standard bracketing analyses was also used to assess the quality of spot measurements and check for any extreme outliers. The δ^18^O measurements that showed primary beam instability or low ^16^O^–^ yield were excluded from the dataset. The final raw SIMS data are presented in S1 Table.

SIMS measures a relative isotopic composition as it is known to be affected by “matrix effects.” Matrix effects refer to a mismatch in chemical compositions and structures between samples being analyzed and standard materials causes an instrumental bias, resulting in a shift in measured values [48–52]. All SIMS δ^18^O_otolith_ measurements were thus calibrated using a PBT-specific offset correction equation developed by Hane et al. [44] to consider sample-standard mismatch effects:

$$IRMS\;\delta^{18}O_{\mathrm{otolith}\;(\mathrm{VPDB})}=SIMS\;\delta^{18}O_{\mathrm{otolith}\;(\mathrm{VPDB})}+0.41$$ (1)

by simply adding 0.41‰, which is an average offset between the SIMS-measured and IRMS-measured δ^18^O_otolith_ values. The SIMS δ^18^O_otolith_ results are reported in standard δ notation (‰) relative to VPDB that have been converted from VSMOW by using the latest published conversion equation ($\delta^{18}O_{\mathrm{VSMOW}}=1.03092\times \delta^{18}O_{\mathrm{VPDB}}+30.92$, [53, 54]).

### Discrimination of the natal origin

To identify discrete groups of individuals that have the same natal origin, an unsupervised cluster analysis using *k-*means algorithm (the cluster package in R, version 3.6.3) was performed on the core δ^18^O_otolith_ values and the distance from the otolith core to the first opaque zone. *K-*means clustering is a centroid-based partitioning clustering approach that partitions observations into discrete clusters by assigning each data point to the nearest cluster using Euclidean distance. This method requires the user to specify the number of clusters before clustering is performed. Although our goal was to identify two distinct groups (the Sea of Japan-born and the Nansei Islands area-born fish), the optimal number of clusters was assessed using *fvis_nbclust* and *NbClust* functions of factoextra and NbClust packages in R, both of which compute the best number of *k* based on 30 different evaluation methods including “the within-cluster sums of squares” (which measures the compactness of each cluster) and “the Silhouette value” (which measures the distance between clusters). The resulting clusters were also evaluated by visual inspection to assess if the clustering results best explained the data.

We used the core δ^18^O_otolith_ values and the distance from the otolith core to the first opaque zone as two clustering variables. The core δ^18^O_otolith_ deposited during the larval stage (less than 20 days post-hatch [DPH]) likely reflects the natal thermal environment of fish because PBT larvae tend to stay in waters within or close to the spawning grounds. The SEM images were used to observe and count the daily growth increments around the core region. We made sure that the δ^18^O_otolith_ values taken from the otolith region deposited within 20 DPH were used as the core δ^18^O_otolith_. The distance from the otolith core to the first opaque zone can be used as an early growth indicator as otolith length of bluefin tuna is highly correlated with fish somatic growth [55, 56]. The preliminary assessment was conducted to evaluate the relationship between the first opaque zone formation in PBT otoliths and the first δ^18^O_otolith_ peak (representing the lowest winter temperature experienced during the first year of life) that was determined using spline-interpolated δ^18^O_otolith_ chronological profiles. There was a strong positive correlation between the location of the first opaque zone and that of the δ^18^O_otolith_ peak (Pearson correlation coefficient, *r* = 0.76, *p* < 0.0001), indicating that the formation of the first opaque zone in PBT otoliths occurs during their first winter. Therefore, the distance from the otolith core to the first opaque zone was shown to be an appropriate variable representing the early growth of PBT until their first winter.

In addition to the samples analyzed in this study, 5 otolith samples analyzed in Hane et al. [44] were included for the cluster analysis to increase the sample size, totaling *n* = 51.

### Temperature reconstructions

Water temperature experienced during the larval stage was reconstructed from the core δ^18^O_otolith_ data obtained by SIMS using a species-specific oxygen isotope fractionation equation. For accurate estimation of ambient water temperature, the oxygen isotope composition of seawater (δ^18^O_seawater_) at the time of otolith formation is needed to correct for the effect of salinity on δ^18^O_otolith_. As there were not adequate δ^18^O_seawater_ data in Japanese waters available at the time of analysis, δ^18^O_seawater_ in each spawning ground was calculated using regional salinity–δ^18^O_seawater_ mixing curves in the Sea of Japan [57] and the East China Sea [58]:

$$\delta^{18}O_{\mathrm{seawater}\;(\mathrm{VSMOW})}=-9.1+0.27\times S\;(the\;Sea\;of\;Japan)$$ (2)

$$\delta^{18}O_{\mathrm{seawater}\;(\mathrm{VSMOW})}=-7.74+0.23\times S\;(the\;East\;China\;Sea)$$ (3)

where δ^18^O_seawater_ is oxygen isotope composition of seawater and *S* is salinity. To account for the interannual variation in salinity experienced among fish of different year-classes, year-specific salinity data were derived from a regional high-resolution ocean model, the Japan Coastal Ocean Prediction Experiment model (JCOPE2, [59, 60]). This model is based on the Princeton Ocean Model [61] that is driven by monthly wind stress, heat flux, and salt flux, and it assimilates a large amount of *in-situ* and satellite-derived data for improved data quality. The JCOPE2 model provides daily fields of temperature, salinity, sea surface height, and velocity with a horizontal resolution of 1/12° (8 to 9 km) and 47 vertical layers. Using year-specific salinity data during the peak spawning months (July in the Sea of Japan, May–June in the Nansei Islands area), the δ^18^O_seawater_ was calculated for each spawning ground for the years 1993–2017. Salinity data at 5 m depth was used as the PBT larvae are mainly found at or near the surface [62]. For temperature reconstruction, the δ^18^O_seawater_ values were converted from the VSMOW scale to the VPDB scale by simply subtracting 0.27‰ [63] as the δ^18^O_otolith_ in the oxygen isotope fractionation equation is measured against the VPDB standard.

Ambient water temperatures experienced during the larval stage were estimated using an oxygen isotope fractionation equation of PBT larvae [34] with a minor modification:

$$\delta^{18}O_{\mathrm{otolith}\;(\mathrm{VPDB})}-\delta^{18}O_{\mathrm{water}\;(\mathrm{VPDB})}=5.243-0.270\times T({}^{\circ}C)$$ (4)

where δ^18^O_otolith_ is the oxygen isotope value of otolith and δ^18^O_water_ is the seawater oxygen isotope composition. The intercept of the original equation ($\delta^{18}O_{\mathrm{otolith}\;[\mathrm{VPDB}]}-\delta^{18}O_{\mathrm{water}\;[\mathrm{VPDB}]}=5.193-0.270\times T$) was slightly modified as the most updated VSMOW–VPDB correction equation was applied on the δ^18^O_seawater_ data used in Kitagawa et al. [34]. The difference in the resulting temperature estimates using the modified equation from the original equation is approximately −0.2°C.

We evaluated the accuracy of absolute temperature reconstruction by comparing the ambient temperature exposure of PBT larvae estimated from the SIMS-measured core δ^18^O_otolith_ values with sea surface temperature (SST, 5 m) in the spawning grounds that were calculated using the JCOPE2 model. As the hatching date of each individual fish was not available, the seasonal SST (the Sea of Japan: July, the Nansei Islands area: May to June) was compared with the reconstructed temperatures averaged over the fish of the same year-class. Due to the low sample size in some year-classes, the comparison was made for the 2009, 2010, and 2011 year-classes. We used the temperature estimates calculated based on the clustering results when *k* = 2.

## Results

### SIMS δ^18^O_otolith_ measurements

**Fig 4:**
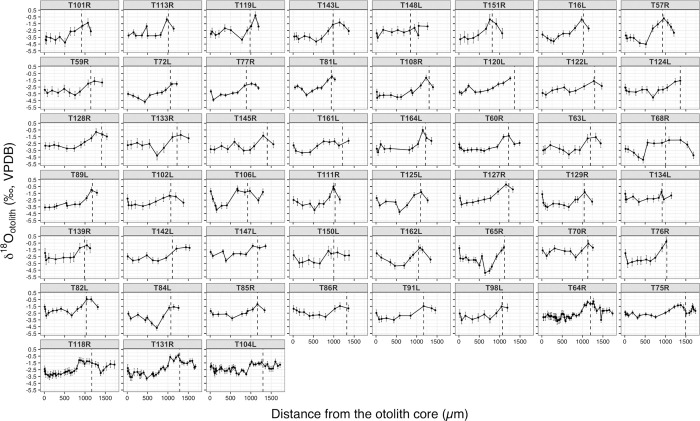
The δ^18^O_otolith_ chronologies recording the early life and immature stages of adult Pacific bluefin tuna. Error bars indicate the spot-to-spot precision of SIMS analyses (± 2SD). Dashed vertical lines indicate the location of the first opaque zone (a dark line-like structure formed in winter) in otoliths corresponding to the lowest temperature experienced by an individual fish in winter.

An increasing trend was observed in the δ^18^O_otolith_ chronological record during the early life and immature stages of all fish samples, with δ^18^O_otolith_ values progressively enriched toward the first opaque zone (Fig 4). The average δ^18^O_otolith_ values from the otolith core to about 500 to 750 μm ranged between –3.8 and –1.2‰ (VPDB), where most fish showed a gradual decrease in δ^18^O_otolith_ values during this period. The δ^18^O_otolith_ values then increased toward the first opaque zone, peaking at –0.2 to –2.1‰ (VPDB).

**Fig 5:**
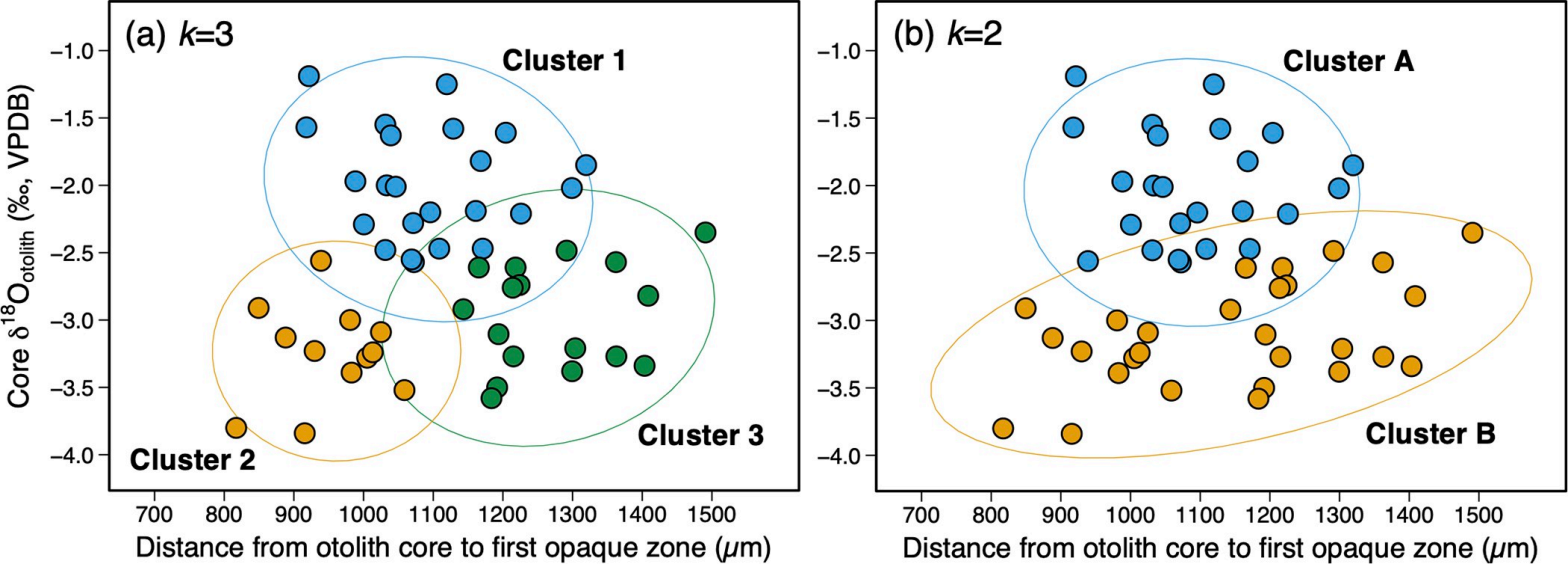
Clustering results on core δ^18^O_otolith_ and the distance from otolith core to first opaque zone. The ellipses show 95% confidence intervals. (a) Clustering results when *k* = 3. Cluster 1 (blue circles) are fish presumed to be born in the Sea of Japan and Clusters 2 (orange circles) and 3 (green circles) are fish presumed to be born in the Nansei Islands area. (b) Clustering results when *k* = 2. Clusters A (blue circles) and B (orange circles) are fish presumed to be born in the Sea of Japan and the Nansei Islands area, respectively.

The core δ^18^O_otolith_ values corresponding to the larval stage greatly varied among the individuals, ranging between –3.8 and –1.2‰ (VPDB) with most of them falling within the range between –3.5 and –1.5‰ (VPDB) (Fig 5). The distance from the otolith core to the first opaque zone also varied among the individuals, ranging from 817 to 1409 μm.

### Cluster analysis

The results of cluster analysis are presented in Fig 5. The optimal number of clusters was determined to be 3 based on the validation indices including minimization of within-cluster sum of squares and maximization of the distance between clusters. The second optimal number of *k* was 2. Clustering results when *k* = 3 and *k* = 2 both grouped individuals into those with enriched core δ^18^O_otolith_ values (lower temperature) and those with depleted core δ^18^O_otolith_ values (higher temperature). When *k =* 3, Cluster 1 (blue plot in Fig 5A, *n* = 22) was composed by fish with relatively enriched core δ^18^O_otolith_ values ranging between –2.6 and –1.2‰ (VPDB), while Cluster 2 (orange plot in Fig 5A, *n* = 12) and Cluster 3 (green plot in Fig 5A, *n* = 17) were composed by fish with relatively depleted core δ^18^O_otolith_ values ranging from –3.8 to –2.6‰ (VPDB) and –3.6 and –2.4‰ (VPDB), respectively. The distance from the otolith core to the first opaque zone ranged approximately between 900–1300 μm for Cluster 1, 800–1100 μm for Cluster 2, and 1100–1500 μm for Cluster 3.

The clustering results when *k* = 2 were similar to the clustering results when *k* = 3, resulting in groups of fish with enriched core δ^18^O_otolith_ values (Cluster A in Fig 5B, *n* = 23) and depleted core δ^18^O_otolith_ values (Cluster B in Fig 5B, *n* = 28). All individuals who were previously assigned to Clusters 2 and 3 were in fact combined to form a single cluster (Cluster B), except for one individual who was assigned to a different cluster (Cluster A). Mean core δ^18^O_otolith_ for Cluster A and Cluster B were –2.0 and –3.1‰ (VPDB), respectively, with Cluster B showing significantly higher δ^18^O_otolith_ values (unpaired two-samples *t*-test, *p* < 0.001). Although not significantly different (Welch’s *t*-test, *p* = 0.17), Cluster B showed a larger variation in the distance from the otolith core to the first opaque zone (800–1500 μm) than that of Cluster A (900–1300 μm) (*F*-test, *p* < 0.01).

### Salinity, δ^18^O_seawater_, and temperature reconstruction

**Fig 6:**
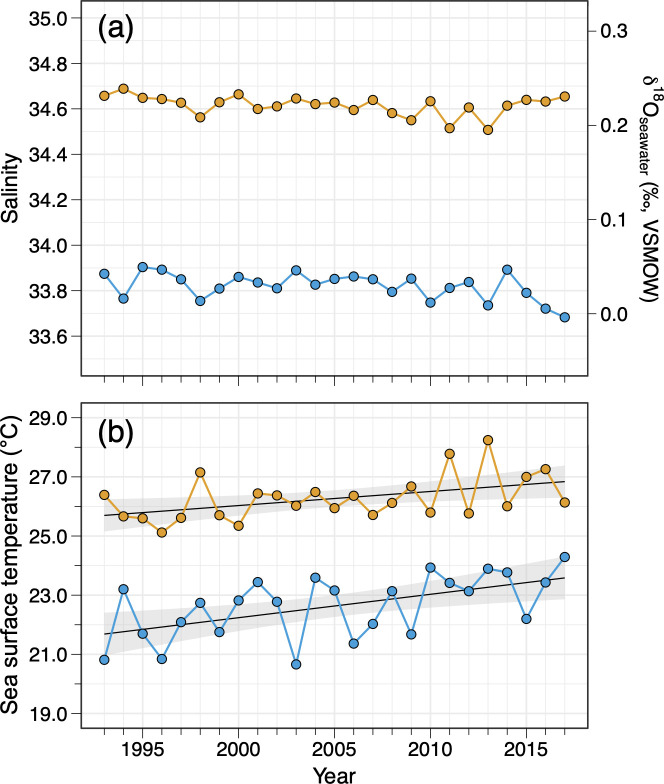
(a) Salinity, δ^18^O_seawater_, and (b) sea surface temperature time series (1993–2017) in the two spawning grounds of Pacific bluefin tuna. Colors represent the Sea of Japan (July: blue plot) and the Nansei Islands area (May–June: orange plot). The δ^18^O_seawater_ values were estimated using salinity data derived from JCOPE2. Grey shaded areas represent the 95% confidence intervals.

The time series data (1993 to 2017) on salinity, δ^18^O_seawater_, and SST are plotted in Fig 6. The average salinity during the peak spawning season of PBT from 1993 to 2017 was 33.82 in the Sea of Japan (July) and 34.58 in the Nansei Islands area (May to June) (Fig 6A). Those values can be converted to δ^18^O_seawater_ values of 0.03‰ (VSMOW) in the Sea of Japan and 0.21‰ (VSMOW) in the Nansei Islands area (Fig 6A), which yields a difference of 0.18‰ (VSMOW) between the two spawning grounds. This 0.18‰ difference is equivalent to 0.67°C in water temperature, indicating that the estimation error of water temperature would be 0.67°C if the natal origin of an individual was assigned incorrectly.

SST during the peak spawning period in the Sea of Japan from 1993 to 2017 was consistently lower than that in the Nansei Islands area (Fig 6B), with a mean difference of 3.6 ± 2.2°C (± 2SD). Spawning temperature of adult PBT have also been reported to be relatively lower in the Sea of Japan than in the Nansei Islands area. We thus assumed fish with enriched core δ^18^O_otolith_ values (Cluster 1 and Cluster A) as those originating from the Sea of Japan and those with depleted core δ^18^O_otolith_ values (Cluster 2, Cluster 3, and Cluster B) as those originating from the Nansei Islands area for the temperature reconstruction (see more detailed interpretation of cluster analysis in Discussion). The mean estimated ambient water temperature (± 2SD) experienced during the larval stage was 25.9 ± 3.1°C for Cluster 1 (the Sea of Japan cluster), 31.1 ± 2.7°C for Cluster 2 (the Nansei Islands area cluster), and 30.3 ± 2.9°C for Cluster 3 (the Nansei Islands area cluster). When *k* = 2, the mean reconstructed temperature was 26.7 ± 3.1°C for Cluster A (the Sea of Japan cluster) and 30.7 ± 2.8°C for Cluster B (the Nansei Islands area cluster).

The average SST (± 2SD) during the spawning season from 2009 to 2011 was 23.0 ± 2.4°C and 26.8 ± 2.0°C in the Sea of Japan and the Nansei Islands area, respectively, giving a SST difference of 3.8 ± 2.0°C between the two regions. The average reconstructed absolute temperatures for the three year-classes (2009, 2010, and 2011) were approximately 3.0 to 4.0°C higher than the corresponding SST, indicating an offset of about 0.8 to 1.1‰ (VPDB) from the SIMS δ^18^O_otolith_ measurements (SIMS δ^18^O_otolith_ yielding lower values). Higher temperature estimates were evident in both clusters, with some individuals experiencing temperatures >30°C (as high as 33.4°C) in the Nansei Islands area cluster. However, the relative temperature difference between Cluster A (the Sea of Japan cluster) and Cluster B (the Nansei Islands area cluster) estimated from the core δ^18^O_otolith_ matched well with that observed in the SST data, which yielded a temperature difference of 3.7 ± 3.2°C (± 2SD) between the two clusters.

## Discussion

Given the negative relationship between δ^18^O_otolith_ and water temperature [29, 33] the observed increasing trend toward the first opaque zone in the δ^18^O_otolith_ chronologies likely reflects a decreasing water temperature experienced during the early life and immature stages of PBT from autumn to winter. Similarly, the initial decrease in δ^18^O_otolith_ is presumed to reflect an increase in water temperature as PBT hatched in the two primary spawning grounds would later be exposed to higher water temperatures in summer, which peaks in late August to early September.

The cluster analysis grouped fish individuals into those experienced relatively higher temperatures (depleted core δ^18^O_otolith_) and those experienced relatively lower temperatures (enriched core δ^18^O_otolith_). Spawning temperatures of PBT in the two spawning grounds have been primarily inferred from the larval surveys [14, 64] and histological analysis of gonads, especially of the ovarian development in female fish [16]. Actively spawning PBT have been found at SSTs between 19.3–27.7°C and 23.9–28.5°C with mean SST values of 23.2°C and 26.7°C in the Sea of Japan and the Nansei Islands area, respectively [14, 16]. PBT larvae have been collected at water temperatures between 18.5–27.9°C and 26.8–29.4°C with mean temperatures of 25.5°C and 28.4°C in the Sea of Japan and the Nansei Islands area, respectively [62]. Although optimal temperature for the PBT larval growth has been reported to be warmer ranging between 24–29°C [65], spawning activity and larvae have been confirmed to occur at cooler temperatures <24°C in the Sea of Japan. The mechanism of spawning at cooler temperatures in the Sea of Japan is still unknown, but smaller and younger individuals seem to predominantly spawn in this area [16]. Also, the Sea of Japan is more productive than the Nansei Islands area, suggesting a selective trade-off between cold water and high prey availability for the larval growth and survival in this area. Overall, spawning occurs at relatively cooler temperatures in the Sea of Japan than in the Nansei Islands area. The δ^18^O_otolith_ enriched- and depleted-groups detected in the clustering results therefore most likely reflect the Sea of Japan born- and the Nansei Islands area born-fish.

The great variation in the core δ^18^O_otolith_ values detected among the individuals indicates that the PBT larvae experienced a wide range of thermal environments. The observed range of the core δ^18^O_otolith_ values (range: 2.0–2.5‰, VPDB) in our samples corresponds roughly to 7–9°C in water temperature, which is in reasonable agreement with the known spawning temperature range in the Sea of Japan and the Nansei Islands area (see the previous paragraph). This variation was not observed in the previous study [39] that used the conventional IRMS to analyze the core δ^18^O_otolith_ of YOY bluefin. The conventional IRMS requires a larger amount of otolith powder compared to SIMS, which essentially limits the spatial and temporal resolution (10 μm for SIMS vs. >250 μm for IRMS). Such coarse resolution of the IRMS results in δ^18^O_otolith_ averaging over the entire early life stages and quite often beyond that of the fish, possibly masking the pronounced difference in the environment recorded during the egg and larval stages. SIMS technique should therefore be considered as the primary method when focusing on reconstructing and examining the early life history, particularly the larval stages of fish.

The larger variation in the distance from the otolith core to the first opaque zone observed for Cluster B with depleted core δ^18^O_otolith_ suggests that spawning continued for a longer period, likely reflecting a longer spawning duration in the Nansei Islands area. Spawning in the Nansei Islands area is reported to occur for 2–3 months between late April to early July [13, 14], whereas that in the Sea of Japan occurs for 1.5 months during July and early August [16, 64]. Given the longer spawning period in the Nansei Islands area, the two resulting clusters (Clusters 2 and 3) may represent the early-born and the late-born fish in this area. This is supported by an increasing trend observed in the core δ^18^O_otolith_ for Cluster B (see Fig 5B, *t*-test, *p* = 0.01), which reflects an increasing water temperature experienced by the PBT larvae throughout the entire spawning season in the Nansei Islands area (note that the longer the otolith distance is, the earlier the fish is born). Shiao et al. [39] found that the nearest distance of the first δ^18^O_otolith_ peak from the otolith core was shorter for the YOY born in the Sea of Japan due to the late spawning season, resulting in a shorter growth period. Our results also showed a similar pattern for Cluster 1 (the Sea of Japan cluster) and Cluster 3 (the early-born Nansei Islands area cluster), except for Cluster 2 (the late-born Nansei Islands area cluster) which had the shortest otolith distance. This might be the result of low productivity environments that are inherent to the nursery areas in the northwestern Pacific [66], which could cause reduced growth during juvenile and YOY stages of PBT. PBT in the Sea of Japan, in contrast, would benefit from higher primary and secondary productivity associated with nutrient-rich cold waters brought up by upwelling [67] for their greater juvenile and YOY growth rates [66], supposedly compensating for their shorter growing period. Using the distance from the otolith core to the first δ^18^O_otolith_ peak (or first opaque zone) alone therefore might possibly misassign individuals to the wrong natal origin, particularly for those born in the later spawning season in the Nansei Islands area. As the samples used in our study are all adult fish with unknown origin, further studies should use larvae or juvenile samples with known origin to evaluate the accuracy of the natal origin assignment by SIMS. Nonetheless, the SIMS-based core δ^18^O_otolith_ is a powerful alternative tool that can provide information about the natal thermal environment, which is critical for determining the natal origin of PBT and other fish species that undergo different temperature environments during the early life stages.

The SIMS-generated δ^18^O_otolith_ values in the present study overestimated absolute water temperatures experienced by fish even after the offset correction of 0.41‰ has been applied. Higher absolute temperature estimates based on the SIMS δ^18^O_otolith_ measurements have also been reported in the previous studies [68, 69] and can be attributed to several factors. One of the major sources of uncertainty contributing to the temperature overestimation or lower δ^18^O_otolith_ is an incomplete correction of instrumental mass fractionation caused by matrix effects [51, 52]. In our SIMS analysis, the δ^18^O_otolith_ values are measured against the UWC-3 standard which is currently known to be the most isotopically and chemically homogeneous calcite standard. There are no reliable aragonite standard materials currently available that match exactly with the complex crystalline structure and composition of otoliths. Due to the inherent nature of the SIMS technique, contaminants from the other oxygen-bearing phases such as organic proteins [70, 71] and water content within the otolith matrix cannot be avoided and consequently bias the resulting isotopic values to be lower [72–74]. Although our SIMS δ^18^O_otolith_ results have been calibrated to consider such effects, the single offset correction applied here may not be sufficient to fully address the natural variability of protein incorporation and other matrix-related bias at different life stages of otoliths. In fact, the presence of relative hydrogen content indicated by the background-corrected ^16^OH^−^/^16^O^−^ ratios was higher for the δ^18^O_otolith_ measurements taken from the otolith core than that taken from the other otolith regions. This suggests more incorporation of organic matter and/or water content during the larval stage and might explain the erroneously high temperature estimates observed for both the Sea of Japan and the Nansei Islands area clusters. Nevertheless, such bias on the resulting δ^18^O_otolith_ values can be assumed to be stable and constant as the δ^18^O_otolith_ measurements were taken from the same life stage with the same analytical conditions [40]. Thus, the relative temperature difference should not be affected significantly and remain intact.

Another potential factor affecting the estimate error in temperature reconstruction is δ^18^O_seawater_ values. We used the seasonal δ^18^O_seawater_ instead of those corresponding to the time of otolith formation as hatching date of individuals was not available. The surface salinity in the Nansei Islands area does not fluctuate significantly throughout the spawning season, whereas that in the Sea of Japan is more subject to change due to freshwater discharge mostly from Changjiang (Yangtze) River in China [57]. The effects of low-salinity water from Changjiang river become pronounced toward August and September in the southwestern part of the Sea of Japan, where salinity often becomes lower reaching 33.0 to 33.5 (−0.19 and −0.06‰ [VSMOW]) during the PBT spawning season in August. If PBT larvae hatched in August, the temperature estimates would result in values slightly to moderately lower (approximately 0.3–0.8°C) than those estimated with seasonal δ^18^O_seawater_. The errors in salinity or δ^18^O_seawater_ estimation in the Sea of Japan could thus be a factor causing the temperature overestimation, but not to a significant degree. It is plausible to conclude that the observed temperature offsets are caused by a combination of a sample-standard mismatch, salinity uncertainty, and SIMS analytical precision.

In this study, the use of the core δ^18^O_otolith_ values was shown to serve as an effective thermal indicator for assigning individual fish to its natal origin. This technique can be further used to quantify the recruitment contribution from each spawning ground and subsequently monitor and evaluate the effects of climate change on spawning and population dynamics of various fish. Based on our clustering results, natal origin of PBT for all year-classes pooled was 45% (2009–2011 year-class specific ranges: 33–50%) in the Sea of Japan and 55% (ranges: 50–67%) in the Nansei Islands area. Previous study that used otolith daily increment counts of small PBT [27] estimated the mean contribution rates to be 24% in the Sea of Japan and 76% in the Nansei Islands area, while the year 1994 (anomalously hot summer) accounted for 40% in the Sea of Japan. Higher density of PBT larvae in the Sea of Japan has also been observed after 2010 compared to before 1990s by the larval survey [62], suggesting a reduced early survival or a possible shrinkage and/or northward shift of spawning activity in the Nansei Islands area toward the Sea of Japan in response to a recent warming climate. The SSTs in the two primary spawning grounds have in fact already increased by approximately 1.7°C in the Sea of Japan and 1.2°C in the Nansei Islands area during the 25 years (1993 to 2017) (Fig 6B), with the Sea of Japan possibly becoming more favorable spawning/natal environment for PBT. Although small sample size of each year-class in this study does not allow us to draw significant conclusions about the actual effects of climate change on the PBT population dynamics, SIMS was proven to be effective in investigating the relative thermal exposure of fish larvae and applicable in fish population dynamics studies. With a larger sample size for each year-class, future studies need to investigate the long-term variability of recruitment contribution and its controlling mechanism to gain an in-depth understanding of the effects of ocean warming on spawning and population dynamics of PBT, which is critical for the future management of this economically important species.

## Supporting information

S1 Table Raw SIMS δ^18^O_otolith_ measurements of otoliths of Pacific bluefin tuna *Thunnus orientalis*. (XLSX)
